# Regulating Innate and Adaptive Immunity for Controlling SIV Infection by 25-Hydroxycholesterol

**DOI:** 10.3389/fimmu.2018.02686

**Published:** 2018-11-21

**Authors:** Tongjin Wu, Feng Ma, Xiuchang Ma, Weizhe Jia, Enxiang Pan, Genhong Cheng, Ling Chen, Caijun Sun

**Affiliations:** ^1^School of Public Health (Shenzhen), Sun Yat-sen University, Guangdong, China; ^2^State Key Laboratory of Respiratory Disease, Guangzhou Institutes of Biomedicine and Health, Chinese Academy of Sciences, Guangzhou, China; ^3^Center for Systems Medicine, Institute of Basic Medical Sciences, Chinese Academy of Medical Sciences & Peking Union Medical College, Beijing, China; ^4^Suzhou Institute of Systems Medicine, Suzhou, China; ^5^School of Life Sciences, Anhui University, Hefei, China; ^6^College of Bioscience and Bioengineering, Hebei University of Science and Technology, Shijiazhuang, China; ^7^Department of Microbiology, Immunology and Molecular Genetics, University of California, Los Angeles, Los Angeles, CA, United States

**Keywords:** HIV/SIV, inflammation, CTL, immunotherapy, 25-HC

## Abstract

Persistent inflammation and extensive immune activation have been associated with HIV-1/SIV pathogenesis. Previously, we reported that cholesterol-25-hydroxylase (CH25H) and its metabolite 25-hydroxycholesterol (25-HC) had a broad antiviral activity in inhibiting Zika, Ebola, and HIV-1 infection. However, the underlying immunological mechanism of CH25H and 25-HC in inhibiting viral infection remains poorly understood. We report here that 25-HC effectively regulates immune responses for controlling viral infection. CH25H expression was interferon-dependent and induced by SIV infection in monkey-derived macrophages and PBMC cells, and 25-HC inhibited SIV infection both in permissive cell lines and primary monkey lymphocytes. 25-HC also strongly inhibited bacterial lipopolysaccharide (LPS)-stimulated inflammation and restricted mitogen-stimulated proliferation in primary monkey lymphocytes. Strikingly, 25-HC promoted SIV-specific IFN-γ-producing cellular responses, but selectively suppressed proinflammatory CD4+ T lymphocytes secreting IL-2 and TNF-α cytokines in vaccinated mice. In addition, 25-HC had no significant immunosuppressive effects on cytotoxic CD8+ T lymphocytes or antibody-producing B lymphocytes. Collectively, 25-HC modulated both innate and adaptive immune responses toward inhibiting HIV/SIV infection. This study provides insights into improving vaccination and immunotherapy regimes against HIV-1 infection.

## Highlights

- The expression of CH25H was induced by interferon stimulation and SIV infection.
- 25-HC strongly inhibited inflammation and restricted mitogen-stimulated proliferation in primary monkey lymphocytes.
- 25-HC promoted the SIV vaccine-elicited antigen-specific IFN-γ-producing cellular responses, but selectively suppressed proinflammatory CD4+ T lymphocytes secreting IL-2 and TNF-α cytokines in vaccinated mice.
- 25-HC had no significant immunosuppressive effects on cytotoxic CD8+ T lymphocytes or antibody-producing B lymphocytes.
- These results demonstrate that 25-HC is beneficial for regulating the inflammation, innate immunity and adaptive immune responses toward inhibiting HIV-1/SIV virus infection.

## Introduction

The human immunodeficiency virus-1 (HIV-1) pandemic continues to be a serious challenge for global health. An effective vaccine or immune modulation for controlling HIV-1 infection would help to the decrease disease burden. Although immune correlates for protection are still under investigation, vaccine candidates that are capable of inducing production of broadly neutralizing antibodies (bnAbs) and/or broad-spectrum cytotoxic T lymphocyte (CTL) responses are desirable for a successful HIV-1 vaccine (1, 2). The production of both bnAbs and CTL responses usually depends on activated CD4+ helper T lymphocytes, which provide signals for proliferation and differentiation of B lymphocytes and CD8+ T lymphocytes (2, 3). However, the role of activated CD4+ T cells in HIV-1 vaccines is controversial because these cells can act as both immune effectors and infection targets for HIV-1 infection (3–5).

HIV-1 patients commonly have chronic inflammation and extensive immune activation, characteristic of the production of inflammatory factors including but not limited to interleukin-1β, interleukin-6, and TNF-α, leading to CD4+ T cell turnover and immune dysfunction (6, 7). Studies have revealed that HIV-1 productive replication occurs predominantly in activated T helper 1 cells (Th1) than in Th2 cells, resulting in decreased percentage of Th1 cells and failure of Th1/Th2 arms balance (8, 9); and this eventually accelerates the clinical progression to AIDS. Moreover, the activated CD4+ T cells in immunized individuals might provide more susceptible targets for HIV-1 acquisition and thus may account for the failure of HIV-1 vaccine candidates like as STEP and HVTN503 (10–13). Therefore, a balance between activation and suppression of CD4+ T cells should be critical for controlling HIV-1 infection and is of great importance for HIV-1 vaccination and immunotherapy.

Cholesterol-25-hydroxylase (CH25H) and its metabolite 25-hydroxycholesterol (25-HC) were recently shown to have broad anti-viral activities, against viruses like Zika, Ebola and HIV-1, by our groups and others (14–16). However, the underlying mechanism of this inhibition is not clear. 25-HC can modulate cholesterol metabolism, for example, by promoting cholesterol transport protein ATP-binding cassette A1 (ABCA1) expression that results in cholesterol reduction in the cytoplasm; this feature might contribute to its broad anti-viral activity by interfering with virus-cell membrane fusion, since a high cholesterol level in cell plasma is required during viral assembly, release and spread (17–20). Moreover, increasing data have demonstrated that CH25H and its metabolite participate in regulating immune responses. CH25H is coded by the family of interferon-stimulated genes (ISG) (15, 21), and involved in innate immunity and anti-inflammasome activation by the sensor protein absent in melanoma 2 (AIM2) against microbial infection (22, 23). In addition, 25-HC plays roles in recruiting monocytes/ macrophages (24), trafficking proinflammatory Th1 lymphocytes (25), and mediating neuroinflammation via activation of NLRP3 inflammasome (26, 27). However, it is not clear how 25-HC affects HIV/SIV-related innate immunity and adaptive T cell functions.

In the present study, our results demonstrated that 25-HC modulated both innate and adaptive immune responses toward inhibiting viral infection. This work is helpful for understanding the immunological mechanism by which CH25H inhibits HIV-1/SIV infection, and provides insights into improvements of immunotherapy against HIV-1 infection.

## Materials and methods

### Cells, virus, and other reagents

Mice bone-marrow-derived macrophages (J2-BMMs) and interferon receptor-deficient (Ifnar1^−/−^) BMMs were kindly provided by Genhong Cheng's laboratory at the University of California, Los Angeles (UCLA). The T-B lymphoblast fusion cell line (174 × CEM) was kindly provided by the NIH AIDS Research and Reference Reagent Program. TZM-bl cells (also called JC53BL-13) were obtained from the NIH AIDS Research and Reference Reagent Program, and are a CXCR4-positive HeLa cell clone engineered to express CD4, CCR5 and Tat-regulated Luc reporter gene. TZM-bl cells are extensively used in the field of HIV/SIV studies, and they are permissive to infection by a wide variety of HIV, SIV and SHIV strains. Mice splenocytes were freshly isolated from C57BL/6 mice (Vital River Laboratories, China), and macaque peripheral blood mononuclear cells (PBMCs) were freshly isolated from Chinese-origin rhesus monkeys bred in our institute (experimental animal center of Guangzhou Institute of Biomedicine and Health (GIBH), Guangzhou, China). Cells were cultured in complete RPMI 1640 medium containing 10% fetal bovine serum (FBS) (Gibco), 10 mM HEPES solution (Gibco), 55 μM β-mercaptoethanol (Gibco), 1 mM sodium pyruvate (Gibco) and 2 mM L-glutamine (Gibco).

Adenovirus serotype 5 (Ad5)-based SIV vaccine (Ad5-SIV Env) expressing the simian immunodeficiency virus 239 strain (SIVmac239) Env structural protein was constructed by our laboratory, and the construction, amplification, identification, purification and titration of this recombinant adenovirus are described in our previous studies (1, 28). The transgene of SIVmac239 env in the recombinant adenovirus is driven by CMV promotor. The titer for Ad- SIV Env was 6.28 × 10^12^ VP/ml and 9.67 × 10^9^ TCID50/ml. Ad5-EGFP, Ad5-Luci and SIVmac239 were propagated, purified and tittered in our lab.

25-hydroxycholesterol was purchased from Sigma, dissolved in ethanol, and stored at −20°C. Recombinant rhesus macaque/cynomolgus IFN-alpha protein, with similar activity to human IFN Alpha 2, was purchased from R&D Systems, Inc. Lipopolysaccharide (LPS) from *Escherichia coli* 0111:B4 was purchased from Sigma. Concanavalin A (ConA, Sigma), ionomycin (Ion, Sigma) and phorbol myristate acetate (PMA, Enzo Biochem, Inc.) were prepared and stored according to the manufacturer's instructions. Peptides of SIVmac239 Env were kindly provided by the NIH AIDS Research and Reference Reagent Program. Peptide pools were dissolved at 0.4 mg/ml in DMSO and stored at −80°C. The monoclonal antibodies and polyclonal antibodies used in this study were purchased from indicated companies as mentioned in the following methods. The siRNA kit for human CH25H was purchased from Dharmacon company (SMARTpool: ON-TARGETplus CH25H siRNA). Anti-CH25H antibody was purchased from Abcam (ab133933), and anti-GAPDH antibody was purchased from Cell Signaling Technology (Danvers, MA).

### Animal experiments and ethics statement

Six-to-eight-week-old C57BL/6 female mice were housed in the experimental animal center of GIBH (Guangzhou, China). The care and use of the mice splenocytes and macaque PBMCs in this study were carried out in accordance with the guidelines of “Regulations for the Administration of Affairs Concerning Experimental Animals” approved by the State Council of People's Republic of China. All animal experimental protocols were approved by the Institutional Animal Care and Use Committee of GIBH. All procedures were performed by trained personnel under the supervision of veterinarians. All mice were purchased from the Vital River Laboratories (Beijing, China). As shown in **Figure 4A**, mice were randomly divided into four groups, each consisting of 10 mice. Then, 1 × 10^9^ viral particles (VP) of Ad-SIV Env were intramuscularly injected at week 0 and week 3. During immunization, mice were intravenously injected daily with the indicated amount of 25-HC. At weeks 3 and 5, five mice from each group were sacrificed and lymphocytes from spleen and bone marrow were isolated for subsequent immunological assays.

### Cell viability assay

Cells were treated with different concentrations of 25-HC to generate dose-based curves. After 24 or 120 h, cell viability was determined using a Cell Counting Kit-8 (Dojindo) according to the manufacturer's instructions. Absorbance was measured using the Mithras LB943 microplate reader (Berthold) at 450 nm. Results are presented as a percentage comparing the absorbance of treated samples with those of respective controls.

### Virus infection inhibition assay

174 × CEM cell lines were pre-treated with 25-HC for 12 h and infected with SIV virus (0.1 MOI). Macaque PBMCs were pre-activated with PHA (Sigma) and IL-2 (Sigma) for 24 h before being incubated with 25-HC, and then infected with SIV virus (0.1 MOI). After 4 h infection, cells were harvested and washed to remove free virus. Then, total RNA was extracted to quantify the SIV Gag gene by RT-PCR. To explore the antiviral ability of 25-HC after the viral entry step, freshly isolated PBMCs from SIV-infected monkey were cultured for 60 h in medium containing 25-HC, and SIV RNA from the culture supernatant was quantified by reverse transcription-PCR (RT-PCR). 25-HC was administered just once at the start of our cell culture experiments.

### Knockdown of CH25H by siRNA

TZM-bl cells were transfected with human CH25H siRNA (SMARTpool: ON-TARGETplus CH25H siRNA, Dharmacon) according to the manufacturer's instructions, and then RT-PCR and western blotting was performed to analyze the level of CH25H expression at 36 h after transfection.

### Quantitative PCR analysis

Total RNA was extracted from cultured cells using a Total RNA Purification Kit (Promega) and reverse-transcribed with iScript cDNA synthesis kit (Bio-Rad). Then, RT-PCR was performed with a QuantiFast SYBR Green PCR Kit (Qiagen) in the CFX-96 Real-time PCR system (Bio-Rad). All gene expression data were normalized to the expression of B2M. SIV RNA from the culture supernatant was purified using the QIAamp Viral RNA Kit (Qiagen), and one-step real-time PCR for quantifying SIV genome copies was performed using the QuantiTect SYBR Green RT-PCR Kit (Qiagen). Viral copy number was calculated based on a standard curve that was prepared from serial dilutions of an *in vitro*-transcribed fragment of the SIVmac239 gag gene as previously described (28). Primers for PCR are listed in Supplementary Table 1.

### Measurement of total cholesterol concentration in mice serum

Total cholesterol was determined based on the COP-PAP method using a Total-cholesterol Assay Kit (Changchun Huili Biotech Co., China). Briefly, 2 μl of serum specimen or standard sample were mixed with 200 μl of reaction substrate and incubated for 15 min at 37°C. Absorbance at 405 nm was measured and cholesterol concentrations were calculated according to the manufacturer's instructions.

### CFSE-based cell proliferation assay

Carboxyfluorescein diacetate succinimidyl ester (CFSE)-based staining for cell proliferation was conducted as previously described (28). Briefly, mice splenocytes or macaque PBMC cells were stained with CFSE and then cultured for 5 days with indicated stimulators in the absence or presence of 25-HC. Cells were harvested and stained with antibody cocktails (anti-CD3-PerCP, anti-CD4-APC and anti-CD8-PE) (BD Pharmingen). Aqua dye (Life Technology) was used to exclude dead cells. Samples were analyzed using the FACSArial instrument (BD) and data were processed with FlowJo 7.6 software (Tree Star, Inc.).

### Multi-color intracellular cytokine staining (ICS) assay

Multi-color ICS was performed as previously reported (29). Briefly, 1 × 10^6^ freshly isolated mouse splenocytes were incubated with peptide pool s for 2 h. Brefeldin A (eBioscience) was added to block cytokine secretion for 8 h. Cells were stained with antibody cocktails (anti-CD3-PerCP, anti-CD4-APC and anti-CD8-FITC) (BD Bioscience) for 30 min at room temperature, permeabilized using Cytofix/Cytoperm buffer (BD Bioscience), and stained with anti-IFN-γ-PE, anti-TNF-α-PE-Cy7, and anti-IL-2-APC-Cy7 (BD Bioscience). Samples were subjected to flow cytometric analysis using the FACSArial instrument and data were processed with FlowJo 7.6 software (Tree Star, Inc.).

### Enzyme-linked immunosorbent spot (ELISPOT) assay

An IFN-γ ELISPOT assay was conducted following previous methods (28, 29). Briefly, MultiScreen 96-well plates (Millipore, Immobilon-P membrane) were coated with anti-mouse IFN-γ monoclonal antibodies (BD Pharmingen) overnight and then blocked with 10% FBS for 2 h at 37°C. Freshly isolated mouse splenocytes were seeded and incubated with peptides for 16 h. After washing, IFN-γ-secreting cells were indicated using a biotinylated anti-mouse IFN-γ polyclonal antibody and HRP-coupled streptomycin (BD Pharmingen) and then colored by the NBT/BCIP reagent (Pierce). Spots were calculated using an ELISPOT reader (Bioreader4000, BIOSYS, Germany), and the data were presented as the number of spot-forming cells (SFC) per million cells.

### IgG-secreting plasma and memory B cell ELISPOT assay

MultiScreen 96-well plates were coated with 20 μg/ml SIVmac239 gp140 proteins (Immune Technology Corp) or 30 μg/ml goat anti-mouse IgG antibodies (Boster). 1 × 10^6^ cells were seeded for the detection of SIV gp140-specific IgG-secreting plasma cells, or 1.5 × 10^5^ cells for the detection of total IgG-secreting plasma cells. After overnight incubation, cells were lysed and probed with HRP-conjugated goat anti-mouse IgG (Southern Biotechnology) and developed with 3-amino-9-ethylcarbazole (Sigma). Spots of antibody-secreting plasma cells (ASC) were counted using an ELISPOT reader and data are presented as the number of ASC per million cells. In some experiments, cells were pre-activated with TLR agonist R848 (invivoGen) and mouse IL-2 (Sigma) for 5 days to detect antibody-secreting memory B cells. The activated B cells were washed and performed ELISPOT assay as above protocol.

### Enzyme-linked immunosorbent assay (ELISA)

The titer of IgG binding antibody for SIVmac239 virus was detected by ELISA as described before (28).

### Data analysis

Flow cytometric data were analyzed using FlowJo version 7.6 software (Tree Star, Inc., Ashland, OR, USA). Statistics and Graphics were generated with GraphPad Prism 5 (GraphPad software Inc., La Jolla, CA, USA) using an unpaired *t*-test, and a two-tailed *p*-value of less than 0.05 was considered statistically significant (^*^P < 0.05, ^**^P < 0.01, ^***^P < 0.001).

## Results

### CH25H expression is interferon-dependent and 25-HC inhibits SIV infection in primary monkey lymphocytes

**Figure 1:**
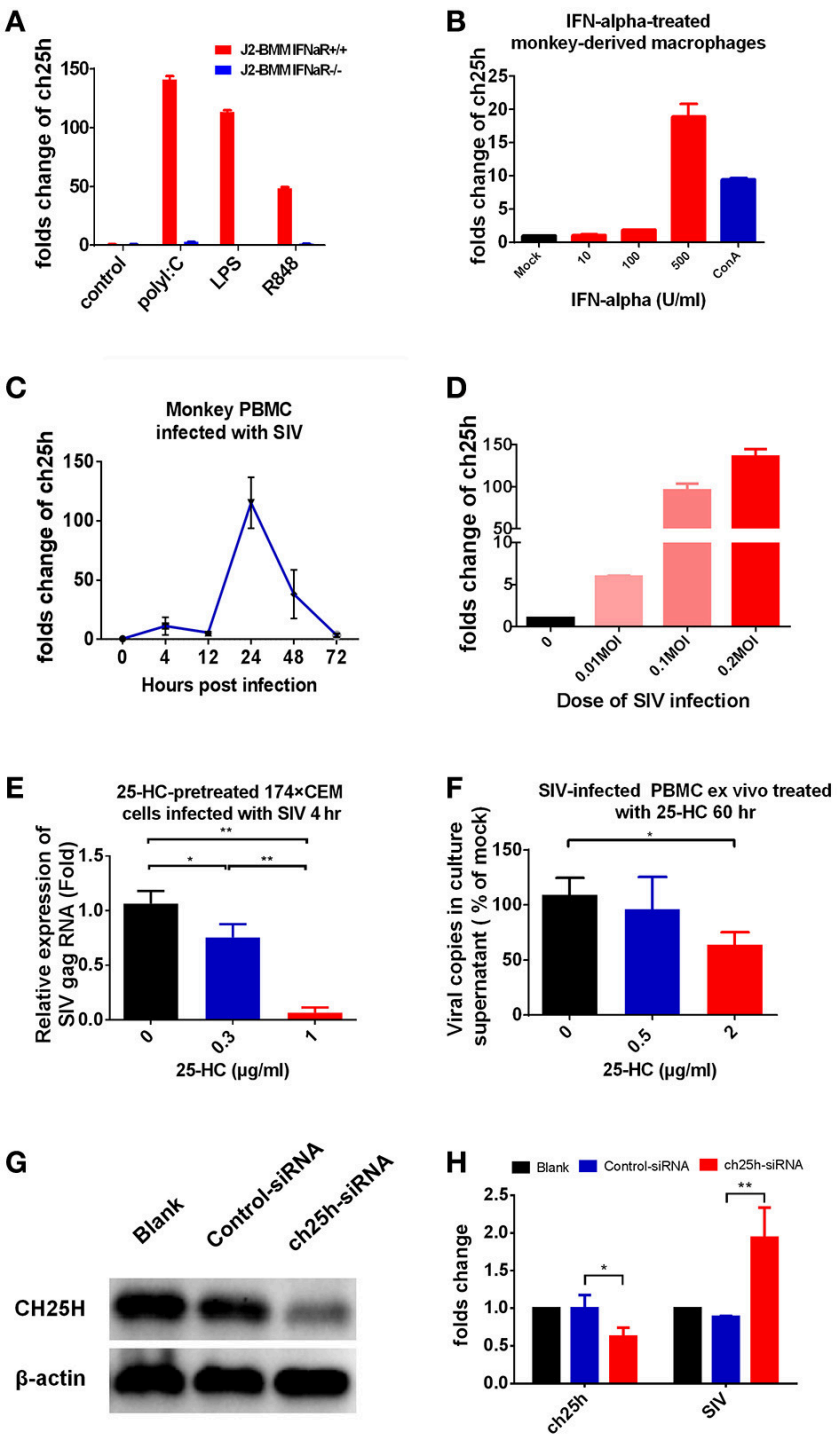
CH25H expression was IFN-dependent and 25-HC inhibited SIV infection in both a permissive cell line and macaque PBMCs. **(A)** Wild-type bone marrow-derived macrophages (J2-BMMs) or interferon receptor-deficient (Ifnar1^−/−^) BMMs from mice were stimulated for 4 h with polyI:C (25 μg/ml), LPS (2 μg/ml) or R848 (1 μg/ml), and then total mRNA was extracted to quantify CH25H expression. **(B)** Monkey macrophage cells, acquired by adherence culture of PBMCs, were treated with the indicated concentration of interferon-α for 12 h, and then total mRNA was extracted to quantify CH25H expression. **(C)** Monkey PBMCs were infected with the SIVmac239 virus (0.1 MOI), and the level of CH25H expression at different time points post-infection was detected by quantitative RT-PCR. **(D)** Monkey PBMCs were infected with different doses of SIVmac239 virus, and the level of CH25H expression was detected by qRT-PCR at 24 h post-infection. **(E)** 174 × CEM cells were pre-treated with different concentrations of 25-HC for 12 h and infected with SIVmac239 virus (0.1 MOI) for 4 h, and then the level of SIV gag expression was detected by qRT-PCR. **(F)** Isolated PBMCs were incubated with or without 25-HC at triplicate wells for 60 h, and the viral particles in culture supernatants were quantified by qRT-PCR. **(G)** TZM-bl cells were transfected with the human CH25H siRNA (SMARTpool: ON-TARGETplus CH25H siRNA, Dharmacon) according to the manufacturer's instructions. At 36 h post-transfection, cell extracts were subjected to western blotting to analyze the level of CH25H protein expression. **(H)** CH25H-knockdown TZM-bl cells were infected with SIVmac239 virus (0.1 MOI) for 4 h, and then the level of CH25H and SIV gag expression was detected by qRT-PCR. The relative numbers of CH25H mRNA or SIVmac239 copies were determined by comparison with the number of B2M mRNA copies. The final data are presented as the mean ± SD of at least triplicate experiments. MOI: multiplicity of infection. ^*^P < 0.05, ^**^P < 0.01.

CH25H gene has been identified as an interferon-stimulated gene (ISG) (15, 21). In this study, we confirmed that CH25H was up-regulated dramatically in response to stimulation by multiple kinds of TLR agonists in wild-type mice bone marrow-derived macrophages (J2-BMMs), and this up-regulation was abrogated in the Ifnar1^−/−^ BMMs (Figure 1A). Moreover, CH25H was up-regulated significantly in macaque macrophages treated with IFN-α (Figure 1B) and in macaque PBMCs infected by SIVmac239 in a dose-dependent manner (Figures 1C,D). Our results supported that CH25H expression is interferon-dependent in mice and non-human primates, and this should be helpful for subsequent studies using mice- or monkey-derived cell samples.

Previously, we found that CH25H and 25-HC inhibited HIV-1 infection (14), but there is no data showing whether they inhibit the simian homologs of HIV-1, SIV, which is commonly used in non-human primate models for studying HIV-1. We observed here that 174 × CEM cells, a permissive cell line for SIV infection and replication, became resistant to SIVmac239 infection after 25-HC treatment in a dose-dependent manner (Figure 1E). Then, we tested whether 25-HC inhibits SIV infection in primary macaque PBMC cells. PBMC cells were isolated from chronically SIVmac239-infected monkeys, which had a high viral load in plasma (4 × 10^5^ copies/ml) in our previous studies (1, 28), and incubated with or without 25-HC (2 μg/ml) for 60 h. Viral copies in culture supernatant of the 25-HC treatment group were significantly reduced, when compared to those of the mock treatment group (Figure 1F, *P* = 0.03). In this study, 4 h post-infection was used for the entry inhibition experiment (Figure 1E), and 60 h post-infection was used for the releasing inhibition experiment (Figure 1F). To further confirm the effect of CH25H on SIV infection, the expression of CH25H in TZM-bl cells was interfered by specific siRNA, and the knockdown efficiency was determined by western blot analysis (Figure 1G) and qRT-PCR (Figure 1H). Our results showed that CH25H-knock down-TZM-bl cells became more susceptible to SIV mac239 infection (Figure 1H).

To clarify whether this observed inhibition was caused by the potential cytotoxicity of 25-HC, we measured the cell viability of the 174 × CEM cell line, mice splenocytes and macaque PBMC cells, when treated with or without 25-HC at different concentrations, by CCK-8 cell proliferation assay. 25-HC showed no apparent cytotoxicity at 3 μg/ml concentrations in our study (Supplementary Figure 1), confirming that 25-HC possesses a direct anti-SIV activity.

In addition, we also detected the interactions between CH25H/25HC and Ad5 infection, since the Ad5-vectored SIV vaccine was to be used in the subsequent animal experiment. Our data showed that 25-HC had no inhibitory effect on Ad5 infection, although Ad5 infection significantly upregulated the level of CH25H expression in 293 cells, A549 cells and Vero cells (Supplementary Figure 2).

### 25-HC attenuates inflammatory responses in LPS- treated monkey PBMCs

**Figure 2:**
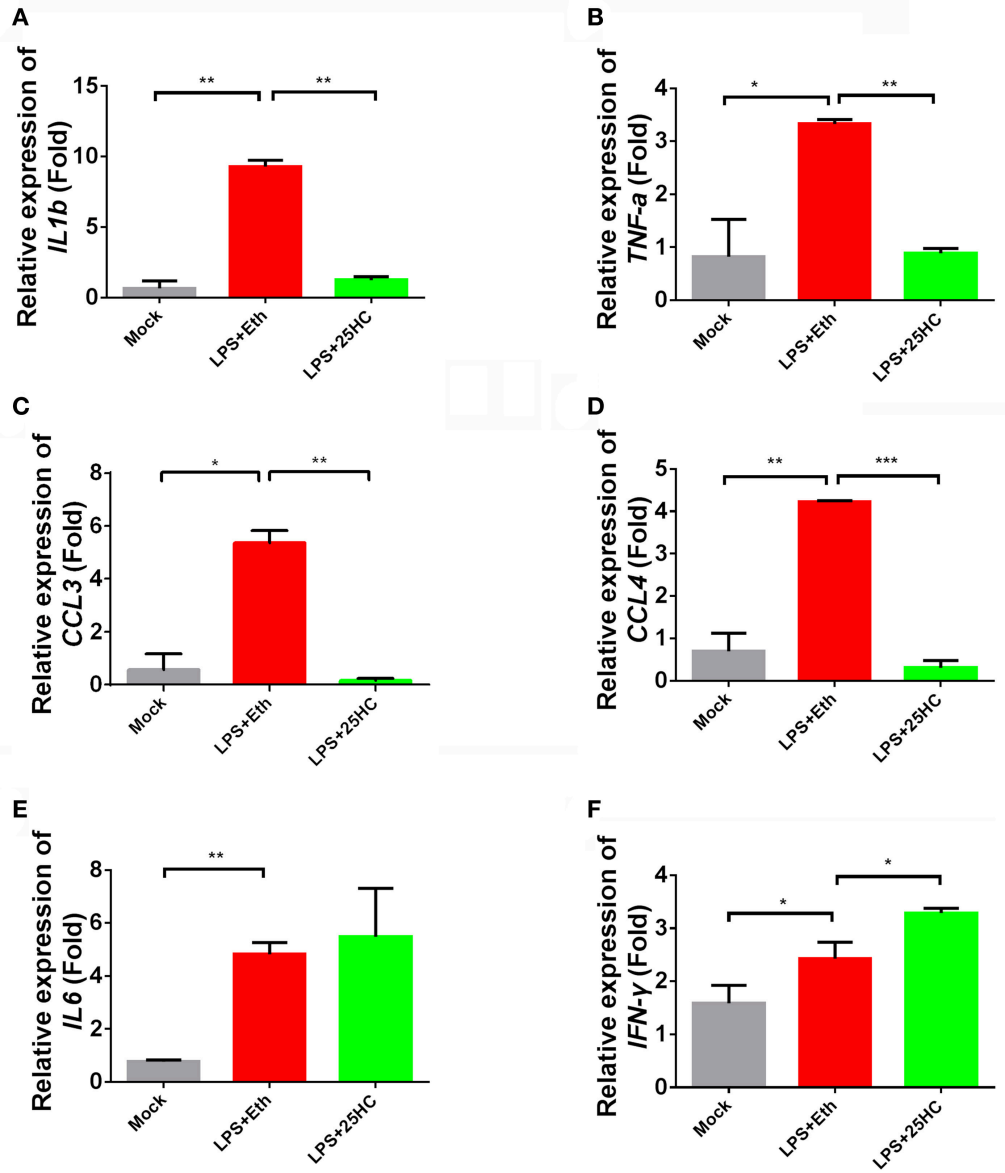
25-HC regulated inflammation responses in macaque PBMCs treated with LPS. Monkey PBMCs were cultured in medium containing 25-HC (0.3 μg/ml), stimulated with LPS (1 μg/ml) for 4 h, and then total mRNA was extracted for the quantification of IL-1β **(A)**, TNF-α **(B)**, CCL3 **(C)**, CCL4 **(D)**, IL-6 **(E)**, and IFN-γ **(F)**. The cells were treated with LPS plus 25-HC concurrently. The gene expression levels were normalized to the expression of B2M, and the data from at least triplicates are shown as the mean ± SD. ^*^P < 0.05, ^**^P < 0.01, ^***^P < 0.001.

A persistent inflammation response, characteristic of the production of inflammatory factors and chemokines like interleukin-1β, TNF-α, interleukin-6, and C-C motif ligand 3 (CCL3), often occurs in chronic HIV-infected patients. Increased circulating LPS caused by intestinal microbial translocation have been associated with chronic inflammation and immune activation in HIV-1 patients (30). To investigate the effects of 25-HC on such inflammatory responses, we used LPS to stimulate freshly isolated macaque PBMC cells in the presence or absence of 25-HC. High expression levels of inflammatory factors and chemokines, including IL-1β, TNF-α, CCL3, CCL4 and IL-6, were induced in LPS-stimulated primary PBMCs (Figures 2A–E). Interestingly, in the presence of 25-HC, inflammation factors, including IL-1β, TNF-α, CCL3 and CCL4, were substantially restored to background levels (Figures 2A–D). In contrast, 25-HC treatment resulted in increased transcription of IFN-γ (Figure 2F), which is an important antiviral cytokine.

### 25-HC restricts proliferation of lymphocytes from mouse splenocytes and macaque PBMCs

**Figure 3:**
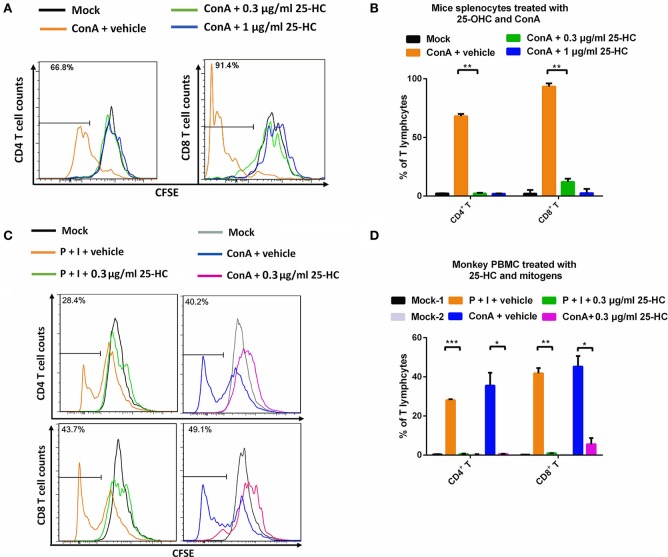
25-HC restricted the proliferation of T lymphocytes in mouse splenocytes and macaque PBMCs. Primary mice splenocytes or monkey PBMC cells were labeled with CFSE and then stimulated for 4–5 days with ConA (1 μg/ml) or a cocktail of PMA (8 ng/ml) and ionomycin (200 ng/ml) as T cell stimulators, with or without 25-HC. These cells were stained with antibodies to label different cell populations and analyzed by flow cytometry. Representative flow cytometry histograms are depicted as the T lymphocyte proliferation of mice splenocytes **(A)** and monkey PBMC cells **(C)**. The frequencies (percentage) of CD4+ T and CD8+ T cell proliferation in mice splenocytes **(B**, *n* = 3**)** and monkey PBMC cells **(D**, *n* = 3**)**. The data were processed with FlowJo software and represented as the mean ± SD. ^*^P < 0.05, ^**^P < 0.01, ^***^P < 0.001.

HIV-1 infection often causes persistent immune activation and excessive lymphocyte proliferation, which is harmful to host immune reconstruction. For example, the abundance of CD4+ T lymphocytes might provide more target cells for HIV-1 infection and transmission. We therefore sought to determine the effects of 25-HC on lymphocyte proliferation. Herein, we used two types of primary cells for this experiment: mice splenocytes and macaque PBMCs. These cells were stimulated with different mitogens, including ConA or a cocktail of PMA and ionomycin as T cell stimulators, and R848 plus IL-2 as a B cell stimulator. These stimulators are sufficient for inducing a high level of T cell or B cell proliferation based on CFSE staining (Figures 3A–C, and Supplementary Figure 3A). Interestingly, when treated with 25-HC, the high level proliferation of T cells, especially CD4+ T cells, was strongly restricted to the initial level prior to stimulation (Figures 3A–D). We also observed decreased proliferation of B cells by 25-HC (Supplementary Figure 3B). These results suggest that 25-HC powerfully suppresses excessive lymphocyte proliferation, which tends to decrease HIV-susceptible targets.

### 25-HC does not cause acute toxicity and cholesterol metabolism change in mice

**Figure 4:**
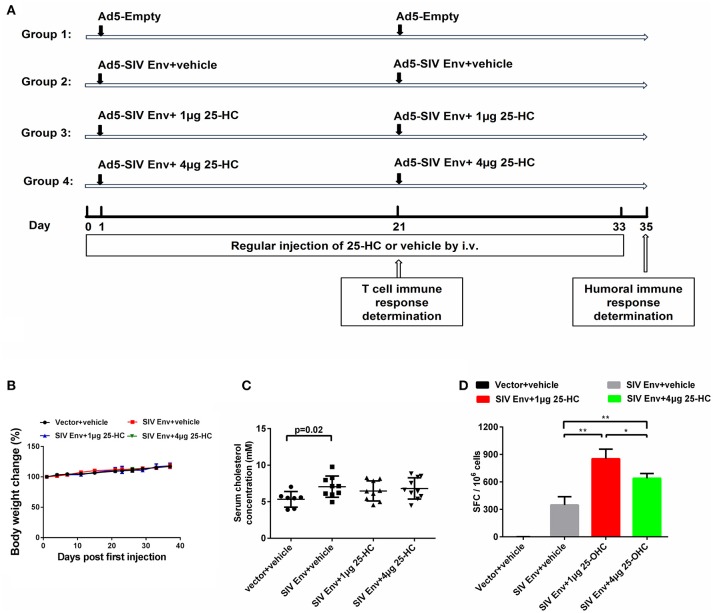
25-HC caused no significant toxicity in mice and promoted HIV-specific IFN-γ-producing immune responses. **(A)** Schedule for evaluating the safety of 25-HC *in vivo* and the effect on immunogenicity of the HIV/SIV vaccine by 25-HC in mice. Briefly, C57BL/6 female mice were randomly divided into four groups, each consisting of 10 mice. A total of 1 × 10^9^ viral particles (VP) of the Ad-based SIV vaccine were intramuscularly injected at weeks 0 and week 3. During the immunization procedure, mice were intravenously injected daily with the indicated amount of 25-HC. At days 21 and 35, five mice from each group were sacrificed, and the splenocytes and bone marrow were harvested and subjected to immunological assays. **(B)** Dynamical changes of mice body weight during the experiment. **(C)** The level of total cholesterol in the serum from immunized mice. **(D)** SIV vaccine-elicited Env antigen-specific cellular immune responses in mice splenocytes were detected using IFN-γ ELISPOT assays following stimulation with Env peptide pools. Data are expressed as the mean ± SD from each group. The final data are representative of two independent mice experiments. SFC: spot-forming cells. ^*^P < 0.05, ^**^P < 0.01.

Since there is very few data available on the safety of *in vivo* administration of 25-HC, we then evaluated its potential toxicity in mice. As described in the methods and Figure 4A, mice were randomly divided into four groups and immunized with HIV-1/SIV vaccine with or without 25-HC. We administrated 25-HC by intravenous injection at two different doses (40 and 160 μg/kg) in this study. Mice treated with 25-HC up to 160 μg/kg showed no significant changes both in their body weight (Figure 4B) and the component of blood cell types as determined from complete blood cell counting (Supplementary Figure 4); also, no other adverse effects were observed. These data suggest that administration of 40 and 160 μg/kg 25-HC did not cause detectable toxicity in mice.

CH25H and 25-HC have been reported to modulate cholesterol metabolism. We therefore examined the level of total cholesterol in serum from immunized mice that received daily intravenous injection of 25-HC. In this study, immunization with adenovirus-based SIV vaccine increased serum cholesterol levels when compared to that of non-immunized mice (Figure 4C, *P* = 0.02). However, there was no statistical change in serum cholesterol levels when mice were continually treated with a high concentration of 25-HC (Figure 4C). These data demonstrate that daily administration of 25-HC up to 160 μg/kg had no obvious adverse effects, and did not significantly change serum cholesterol levels in mice.

### 25-HC promotes SIV antigen-specific IFN-γ-producing cells in mice

We next studied whether 25-HC could regulate immune responses in mice, especially the antigen-specific cellular immune responses which are critical for controlling HIV-1/SIV infection and development of an HIV-1/SIV vaccine. SIV vaccine-elicited antigen-specific cellular immune responses in mice were detected using IFN-γ ELISPOT assays. The frequency of IFN-γ-secreting cells against Env peptides in the vaccine plus 25-HC (40 μg/kg) group was significantly higher compared to the vaccine alone group (Figure 4D). This observation was consistent with our *in vitro* data (Figure 2F). These IFN-γ-mediated immune responses were not further enhanced by treatment with a higher concentration of 25-HC (160 μg/kg) (Figure 4D). Taken together, these data suggest that administration of 25-HC during immunization could enhance total IFN-γ expression and might contribute to their broad anti-viral function.

### 25-HC selectively attenuates proinflammatory Th1 cells secreting IL-2 and TNF-α in mice

**Figure 5:**
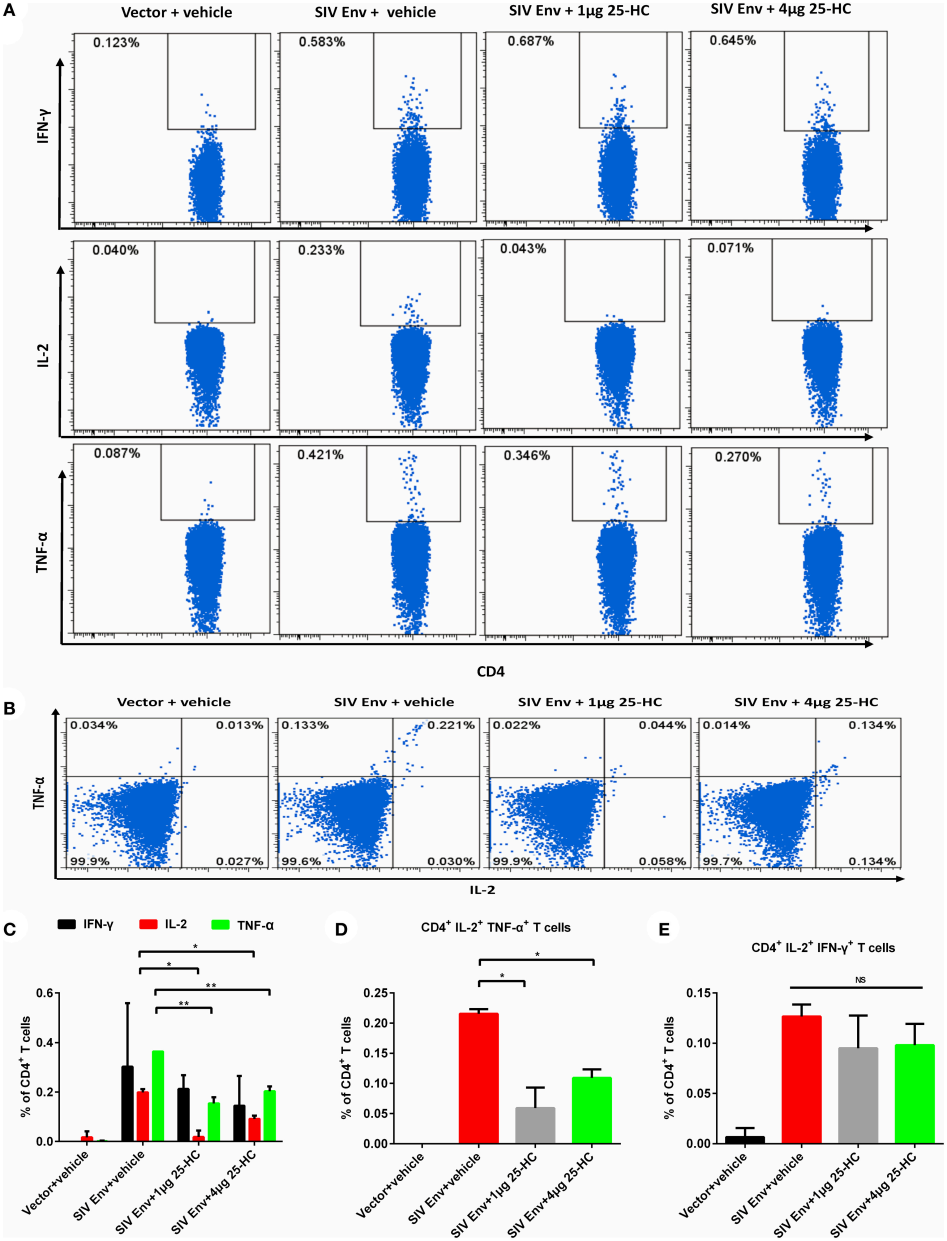
25-HC selectively suppressed antigen-specific CD4+ T cells secreting IL-2 and/or TNF-α cytokines in mice. Mice were immunized as described in Figure 4A, and splenocytes were obtained as described in the Materials and Methods. A total of 500,000 cells were acquired and analyzed by the FACSAria instrument using FlowJo software. **(A,B)** Representative FACS plots to analyze the frequency of cytokine-positive CD4+ T cells in this study. Column graphs represent statistical data for depicting subpopulations of single or double cytokine-positive CD4+ T cells **(C–E)** secreting the cytokines IFN-γ, TNF-α, and IL-2. Data are expressed as the mean ± SD. The representative data shown here were obtained from two independent experiments from 8 to 10 mice for each group. ^*^P < 0.05, ^**^P < 0.01.

To assess how 25-HC affects antigen-specific CD4+ T and CD8+ T cells, splenocytes were isolated from immunized mice, and evaluated for functional T cellular responses using multi-parameter intracellular cytokine staining (ICS). CD4+ T or CD8+ T cell subsets producing one or more cytokines (IFN-γ, TNF-α, and IL-2) were analyzed using the depicted gating strategy (Supplementary Figure 5A). The vaccine effectively induced SIV Env-specific cellular immune responses, characterized by production of CD4+ T and CD8+ T cell subsets secreting functional cytokine(s), either IFN-γ alone, TNF-α alone, IL-2 alone, or dual IFN-γ/TNF-α, or dual IFN-γ/ IL-2 (Figure 5 and Supplementary Figure 5). Notably, the frequency of CD4+ T cells secreting IL-2, TNF-α, and dual IL-2/TNF-α cytokines in the vaccine plus 25-HC (40 μg/kg) group was lower than that of the vaccine alone group (Figures 5C,D). However, no difference was observed in the frequency of IFN-γ-positive CD4+ T cells between the vaccine plus 25-HC group and the vaccine alone group in any conditions (Figures 5C–E). In addition, no difference was observed in the frequency of cytokine-positive CD8+ T cells, including IFN-γ, TNF-α and IL-2 (Supplementary Figures 4B–D). Our results demonstrate that 25-HC can selectively suppress the proinflammatory responses of Th1 cells, especially CD4+ T cells secreting TNF-α and IL-2 *in vivo*.

### 25-HC scarcely affects function of antibody-producing B lymphocytes

**Figure 6:**
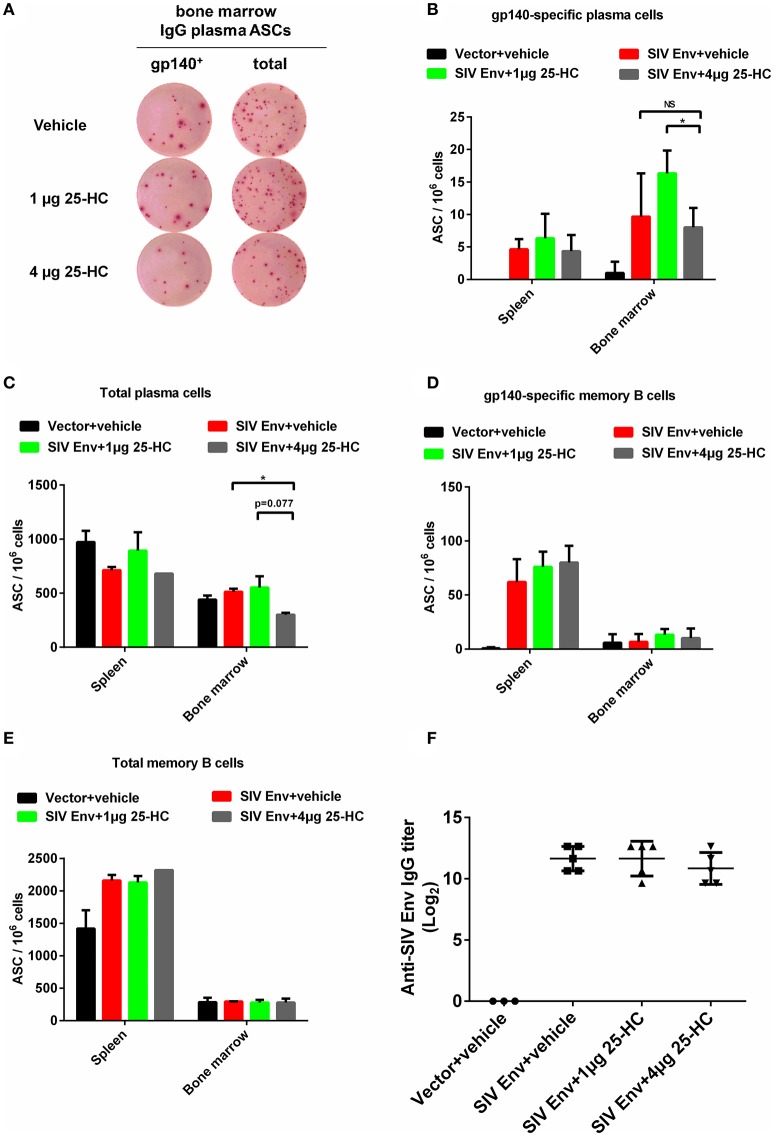
25-HC scarcely affected antibody-producing plasma B lymphocytes and memory B lymphocytes in mice. Mice were immunized as described in Figure 4A, and splenocytes and bone marrow cells were obtained for B cell ELISPOT assay as described in the Materials and Methods. **(A)** A representative result of detection of IgG-producing plasma cells by B cell ELISPOT assay. Column graphs representing statistical data for depicting the frequency of **(B)** gp140-specific IgG ASC plasma cells, **(C)** total IgG ASC plasma cells, **(D)** gp140-specific IgG ASC memory B cells, and **(E)** total IgG ASC memory B cells. **(F)** SIV-binding IgG titers in mice serum assessed by ELISA. Data are expressed as the mean ± SD. The representative data shown here were obtained from two independent experiments. ^*^P < 0.05.

In addition to T cellular immune responses, antigen-specific humoral immunity, including B lymphocytes secreting antibodies with neutralizing activity and antibody-dependent cell-mediated cytotoxicity (ADCC) activity, is essential for preventing and controlling HIV-1 infection. We therefore examined how 25-HC affects the function of antibody-producing plasma B lymphocytes and memory B lymphocytes in mice. IgG antibody-secreting cells (ASC) from splenocytes or bone marrow cells in immunized mice were measured using the B cell ELISPOT assay (Figure 6A). The frequency of total IgG antibody-secreting plasma cells and gp140-specific plasma cells did not significantly change when treated with 25-HC (Figures 6B,C), although a slight decrease of plasma cells was seen in the bone marrow of mice treated with a higher concentration of 25-HC (Figure 6C). Also, a similar frequency of both gp140-specific memory B cells (Figure 6D) and total IgG-secreting memory B cells (Figure 6E) was seen with or without 25-HC treatment in mice. This was confirmed by observing maintenance of antigen-specific-binding IgG antibodies in mice serum of each group (Figure 6F). These data indicate that 25-HC has no significant immunosuppressive effect on the function of antibody-producing plasma B lymphocytes or memory B lymphocytes in mice.

## Discussion

25-HC has been identified as an important antiviral molecule, partly via its ability to inhibit cholesterol biosynthesis. Manipulation of cellular cholesterol content is an important process in host-virus interactions. The lipid profile is altered in HIV-1 patients, and dyslipidemia has also been observed in patients undergoing long-term antiretroviral therapy (31). For example, compared to the healthy population, there were higher triglycerides and total cholesterol and lower high-density lipoprotein (HDL)-cholesterol levels in HIV-1 patients (32), resulting in an increased incidence of cardiovascular events. Studies also revealed that the assembly, budding and release of HIV-1 virus particles preferentially occur in cholesterol-enriched micro-domains (“lipid rafts”) of the plasma membrane (33), and there was enhanced cholesterol metabolism and lower cholesterol levels in HIV non-progressors (34). Interestingly, we found in this study that mice immunized with an adenovirus-based HIV/SIV vaccine increased the serum cholesterol level in comparison to non-immunized mice (Figure 4C), a similar phenomenon documented by a previous report showing that a change in serum lipid profile occurred after influenza vaccination (35). Further studies might be required to verify whether this elevated cholesterol is general for all types of immunizations. The findings of these further studies will provide new approaches for preventing or controlling HIV-1 infection by regulating cholesterol metabolism.

In addition to regulating cholesterol homeostasis, there is increasing data supporting that CH25H and 25-HC have other mechanisms for antiviral activity. First, it was demonstrated that CH25H can inhibit infection and replication of enveloped hepatitis C virus (HCV) through an alternative hydroxylase-independent antiviral mechanism (36). CH25H mutants, with histidine to glutamine mutations at codon positions 242 and 243 in human CH25H could still exhibit anti-HCV activity despite lacking hydroxylase activity. Second, CH25H and its metabolites (25-HC and 27-HC) had an expanded broad antiviral spectrum against several non-enveloped viruses, including human papillomavirus-16 (HPV-16), human rotavirus (HRoV), and human rhinovirus (HRhV) (37). Third, most of the above data were extrapolated from cell lines *in vitro*, and it is unclear whether 25-HC plays a physiological role in regulating cholesterol metabolism *in vivo*. In fact, CD25H-deficient mice exhibited intact cholesterol metabolism compared to that of wild-type mice (38). Moreover, no significant change in serum total cholesterol levels was observed in immunized mice with a high concentration of 25-HC treatment (Figure 4C). Alternatively, recent evidence suggested that 25-HC might act as an important regulator in innate and adaptive immunity (39).

Persistent inflammation and immune activation usually occurs in chronic HIV patients, so anti-inflammation therapy is thought to be helpful for immune reconstitution. Previously, it was debated whether 25-HC represses or augments inflammatory cytokine production. For example, one study showed that 25-HC decreased the production of cytokines from the interleukin-1 family as well as inflammasome activity (22). However, another study found that 25-HC augmented the macrophage and epithelial cell secretion of inflammatory cytokines, such as IL-6, IL-8, and macrophage colony-stimulating factor (M-CSF) (23, 40). In this study, we demonstrated that a low concentration (0.3 μg/ml) of 25-HC dramatically attenuated the inflammatory response in primary monkey PBMCs that were treated with bacterial LPS, a stimulator causing chronic inflammation in HIV-infected patients due to intestinal microbial translocation (30). We also observed that the anti-inflammatory effect of 25-HC decreased at a higher concentration (Data not shown). Therefore, 25-HC regulated inflammation responses through a complex mechanism at different concentrations. Further studies are needed to explore how 25-HC could control inflammation in HIV patients and other autoimmune-related diseases.

In this study, we found for the first time that 25-HC had the capacity to strongly restrict the bulk proliferation of Th1 and B cells. In our study, there was no observed cytotoxicity of 25-HC (0–3 μg/ml) on both mice splenocytes and monkey PBMCs (Supplementary Figure 1B). Therefore, the mechanism of proliferation restriction should not be relevant to cytotoxic effects. Meanwhile, 25-HC has been clearly shown to inhibit the proliferation of T lymphocytes stimulated by mitogens ConA (Figure 3B), while only partially restricting that of B lymphocytes stimulated with TLR agonist R848 plus IL-2 (Supplementary Figure 3). These findings indicate that different cell subtypes (e.g., T cells vs. B cells) may possess heterogeneous resistance to the anti-proliferation property of 25-HC due to the inherent barrier difference of cell proliferation, as well as the cross-talk of signaling pathways driven by 25-HC and stimuli which might contribute to the observed differences. Another possible reason to restrict proliferation might be that cells in the proliferating stage require large amounts of cholesterol, an important component of cell membrane, but 25-HC is reported to negatively regulate the synthesis of cholesterol. Transcriptional profiling of various cell populations may also help explicate these findings. Future work should focus on elucidating the underlying mechanism of proliferation restriction.

Another remarkable observation in this study is that 25-HC improved the total vaccine-elicited antigen-specific IFN-γ-producing cellular responses (Figure 4D), but selectively suppressed proinflammatory responses of TNF-α/IL-2-producing antigen-specific CD4+ T cells (Figure 5 and Supplementary Figure 5). We still do not know the exact mechanism by which CD4+ T cells that secrete TNF-α and IL-2 cytokines (a subset of proinflammatory T cells) are more susceptible to responding to 25-HC *in vivo*, in contrast to cytotoxic CD8+ T cells and B lymphocytes. It might be related to the suppression of inflammatory responses by 25-HC. The host immune system including CD4+ T cells is extensively activated by HIV/SIV infection and vaccination. However, HIV-1 preferentially infects the activated CD4+ T cells (2, 4, 5, 12, 13). Consequently, the activated CD4+ T cells might provide more susceptible targets for HIV-1 transmission instead of protection. In addition, a chronic lymphocytic choriomeningitis virus (LCMV)-infected mouse model has revealed that a CD4+ T-targeting vaccine, in the absence of CD8+ T responses, induced a robust cytokine storm and global inflammation status (41). These findings suggested that a balance between detrimental CD4+ T cell response and protective CD8+ T cell maintenance should be seriously considered when advancing any T cell-based HIV-1 vaccines into clinical trials. Interestingly, we demonstrated the feasibility of attenuating proinflammatory CD4+ T cell function without affecting CD8+ T cell functions.

In addition, our results show that 25-HC improved the total antigen-specific IFN-γ-producing cellular responses detected by ELISPOT assay (Figure 4D), but had no significant effect on IFN-γ-secreting T lymphocytes (Figure 5). Such inconsistency in results could be attributed to the different assays used to detect different immune responses. The ELISPOT assay (Figure 4D) represents the immune responses of whole splenocytes, which are a mixture including not only T lymphocytes, B lymphocytes, but also some monocytes, DC cells, macrophages, and NK cells. These DC cells, macrophages and NK cells, as well as subpopulations of B cells, can also secrete IFN-γ cytokines. In contrast, the FACS-based ICS assay (Figure 5) represents the immune responses of different cell populations which are gated by cell-population markers, such as CD4+ T lymphocytes and CD8+ T lymphocytes. Taking the above analyses together, it may have been possible that 25-HC improved the total antigen-specific IFN-γ-producing cellular responses, especially the IFN-γ-secreting ability of non-lymphocytes (DC, macrophages and monocytes), but had no significant effect on IFN-γ-secreting T lymphocytes.

In conclusion, this work showed 25-HC as a crucial factor to modulate the innate and adaptive immunity toward controlling viral infection. 25-HC improved the overall IFN-γ-producing cellular responses which are beneficial for controlling HIV-1 replication, but selectively suppressed IL-2/TNF-α-producing proinflammatory CD4+ T cells which are helpful for reducing inflammation and infection targets of HIV-1. This study contributes to explaining the anti-HIV-1 activity of 25-HC and provides insights into the development of novel immunotherapy strategies targeting the host immune system against HIV-1 infection and other relevant diseases.

## Author contributions

CS and TW: conceived and designed the experiments. TW, FM, XM, WJ, and EP: performed the experiments. CS and TW: analyzed the data. LC, FM, and GC: contributed reagents, materials. CS, LC and TW: wrote the manuscript. All of author read the final manuscript.

### Conflict of interest statement

The authors declare that the research was conducted in the absence of any commercial or financial relationships that could be construed as a potential conflict of interest.

## Supplementary material

Supplementary Figure 1 Cytotoxic effects of 25-HC on the 174 × CEM cell line and primary cells. The 174 × CEM cell line, mice splenocytes, or monkey PBMC cells were seeded at a density of 5 × 10^5^ cells/well in 48-well plates and treated with different concentrations of 25-HC. The cell viability was measured using the CCK-8 cell proliferation kit after 24 h **(A)** or 120 h **(B)** post-treatment. Absorbance was measured at 450 nm with 650 nm as reference wavelength, and the results are presented as a percentage comparing the absorbance of treated samples and control cells.

Supplementary Figure 2 25HC had no inhibitory effect on Ad5 infection. 293 cells **(A)**, A549 cells **(B)** and Vero cells **(C)** were infected with Ad5-Luci (0.1 MOI), and the level of CH25H expression was detected by qRT-PCR at 24 h post-infection. To evaluate whether adenovirus is susceptible to 25HC treatment, 293 cells **(D)**, A549 cells **(E)** and Vero cells **(F)** were pre-treated with different concentrations of 25-HC for 12 h and infected with Ad5-Luci (0.1 MOI) for 24 h, and then the level of luciferase expression was measured.

Supplementary Figure 3 25-HC inhibited mitogen-driven B cell proliferation. **(A)** CFSE-labeled mice B220^+^ B cells were cultured in conditional medium containing 1 μg/ml R848 and 100 U/ml IL-2 with or without 25-HC for 3 days, and then stained with antibodies for analysis by flow cytometry. **(B)** The corresponding proliferative frequency of mitogen-driven B cells, with data processed by FlowJo software and represented as the mean ± SD. ^*^*P* < 0.05, ^**^*P* < 0.01, ^***^*P* < 0.001.

Supplementary Figure 4 No alteration of the component or proportion of various cell types in mice whole blood by administration of 25-HC. Ten days after the first injection of 25-HC, mice blood was collected in EDTA anticoagulation tubes, and a complete blood cell counting test was performed. The number of white blood cells (WBC) **(A)**, percentage (represented with % value) of lymphocytes **(B)**, neutrophils **(C)**, and monocytes **(D)** are shown, respectively. Data are representative of two independent mice experiments.

Supplementary Figure 5 25-HC caused no functional changes of antigen-specific CD8^+^ T cells. Corresponding to Figure 5, splenocytes were obtained from five mice in each group (Figure 4A) and stained for intracellular cytokines staining (ICS) assay as described in Methods. **(A)** A total of 500,000 cells were acquired and processed using FlowJo software to analyze the cytokine-expressing T lymphocytes. Frequencies of functional CD8^+^ T cell populations secreting IFN-γ, IL-2, or TNF-α cytokine alone **(B)**, as well as dual TNF-α/IL-2 cytokines **(C)** or IFN-γ/IL-2 **(D)** are shown. The representative data shown here were obtained from two independent experiments. ^*^*P* < 0.05, ^**^*P* < 0.01, ^***^*P* < 0.001.

Supplementary Table 1 Primer sets for qRT-PCR. M, mice; S, simian; H, human; Fp, forward primer; Rp, reverse primer.
