# Supplementary figures and images for: Regulating Innate and Adaptive Immunity for Controlling SIV Infection by 25-Hydroxycholesterol
Source: Front Immunol. 2018 Nov 21;9:2686. doi: 10.3389/fimmu.2018.02686 (PMC6262225; doi:10.3389/fimmu.2018.02686)

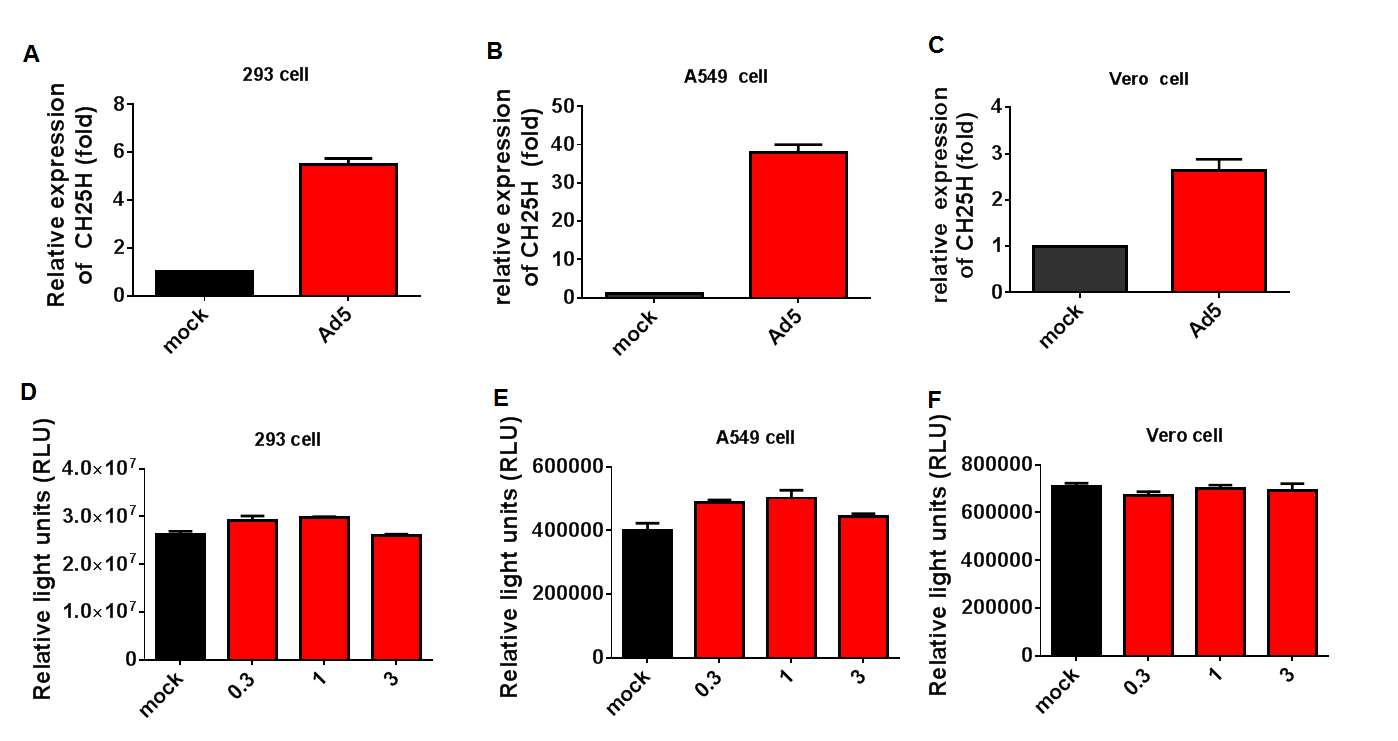

Supplement: Supplementary Figure 2 — 25HC had no inhibitory effect on Ad5 infection. 293 cells (A), A549 cells (B) and Vero cells (C) were infected with Ad5-Luci (0.1 MOI), and the level of CH25H expression was detected by qRT-PCR at 24 h post-infection. To evaluate whether adenovirus is susceptible to 25HC treatment, 293 cells (D), A549 cells (E) and Vero cells (F) were pre-treated with different concentrations of 25-HC for 12 h and infected with Ad5-Luci (0.1 MOI) for 24 h, and then the level of luciferase expression was measured. [file Image_2.TIF]
