# Supplementary material for: Regulating Innate and Adaptive Immunity for Controlling SIV Infection by 25-Hydroxycholesterol
Source: Front Immunol. 2018 Nov 21;9:2686. doi: 10.3389/fimmu.2018.02686 (PMC6262225; doi:10.3389/fimmu.2018.02686)
Supplement: Supplementary Table 1 — Primer sets for qRT-PCR. M, mice; S, simian; H, human; Fp, forward primer; Rp, reverse primer. [file Table_1.docx]

**Supplementary Table 1. Primer sets for qRT-PCR**

| Gene | Primer | Sequence |
| --- | --- | --- |
| *Ch25h* | M-ch25h-Fp | CTGGGACACCATAAGGACAAGG |
|  | M-ch25-Rp | GGGACCCAGGGATACAGGAC |
| *CH25H* | S-ch25h-Fp | CGACATGATGAACGTCACGC |
|  | S-ch25h-Rp | CACCAGTCTGTGAGTGGACC |
| *SIV Gag* | S-g238-Fp | AATACTGTCTGCGTCATCTGG |
|  | S-g382-Rp | ATGGTGCTGTTGGTCTACTTG |
| *B2M* | H-B2M-Fp | GGAAAGCCAAATTTCCTGAATG |
|  | H-B2M-Rp | TGCTGAAAGACAAGTCTGAATGC |
| *TNFA* | S-TNFA-Fp | GAGCACTGAAAGCATGATCCG |
|  | S-TNFA-Rp | GGAGAAGAGGCTGAGGAACCA |
| *IL6* | S-IL6-Fp | CCAGTACTCCCAGGAGAAGATTC |
|  | S-IL6-Rp | GTCGAGGATGTACCGAATGTGT |
| *IL1B* | S-IL1B-Fp | ACGTCGATGGCCCTAAACAG |
|  | S-IL1B-Rp | AAGCCCTCGTTGTAGTGCTC |
| *IFNG* | S-IFNG-Fp | AGAGTGTGGAGACCATCAAGGA |
|  | S-IFNG-Rp | TGCGTTGGACATTCGAGTCAG |
| *CCL3* | S-CCL3-Fp | AGCCCGGTGTCATCTTCCTA |
|  | S-CCL3-Rp | CAGGCACTCAGCTCTAGGTC |
| *CCL4* | S-CCL4-Fp | TTACTACGAGACCAGCAGCCT |
|  | S-CCL4-Rp | AGTTCAGTTCCAGGTCATTAACAT |

M: mice; S: simian; H: human; Fp: forward primer; Rp: reverse primer.
